# Adsorption of Basic Yellow 28 and Basic Blue 3 Dyes from Aqueous Solution Using Silybum Marianum Stem as a Low-Cost Adsorbent

**DOI:** 10.3390/molecules28186639

**Published:** 2023-09-15

**Authors:** Türkan Börklü Budak

**Affiliations:** Department of Chemistry, Faculty of Art and Science, Yildiz Technical University, 34220 Istanbul, Turkey; tborklu@yildiz.edu.tr or turkanborklu@yahoo.com

**Keywords:** adsorption, basic yellow 28, basic blue 3, silybum marianum stem

## Abstract

In the present study, the ability of an adsorbent (SLM Stem) obtained from the stem of the *Silybum Marianum* plant to treat wastewater containing the cationic dyes basic blue 3 (BB3) and basic yellow 28 (BY28) from aqueous solutions was investigated using a batch method. Then, the SLM Stem (SLM Stem-Natural) adsorbent was carbonized at different temperatures (200–900 °C) and the removal capacity of the products obtained for both dyes was examined again. The investigation continued with the product carbonized at 800 °C (SLM Stem-800 °C), the adsorbent with the highest removal capacity. The dyestuff removal studies were continued with the SLM Stem-Natural and SLM Stem-800 °C adsorbents because they had the highest removal values. The surface properties of these two adsorbents were investigated using IR, SEM, and XRD measurements. It was determined that the SLM Stem-Natural has mainly non-porous material, and the SLM Stem-800 °C has a microporous structure. The optimal values for various parameters, including adsorbent amount, initial dye solution concentration, contact time, temperature, pH, and agitation speed, were investigated for BY28 dye and were 0.05 g, 15 mg/L, 30 min, 40 °C, pH 6 and 100 rpm when SLM Stem-Natural adsorbent was used and, 0.15 g, 30 mg/L, 30 min, 40 °C, pH 10, and 150 rpm when SLM Stem-800 °C adsorbent was used. For BB3 dye, optimal parameter values of 0.20 g, 10 mg/L, 30 min, 25 °C, pH 7, and 100 rpm were obtained when SLM Stem-Natural adsorbent was used and 0.15 g, 15 mg/L, 40 min, 40 °C, pH 10, and 100 rpm when SLM Stem-800 °C adsorbent was used. The Langmuir isotherm described the adsorption process best, with a value of r^2^ = 0.9987. When SLM Stem-800 °C adsorbent was used for BY28 dye at 25 °C, the highest q_m_ value in the Langmuir isotherm was 271.73 mg/g. When the study was repeated with actual water samples under optimum conditions, the highest removal for the BY28 dye was 99.9% in tap water with the SLM Stem-800 °C adsorbent. Furthermore, the reuse study showed the adsorbent’s efficiency even after three repetitions.

## 1. Introduction

Nowadays, studies on the protection of natural ecosystems are gaining importance. Increasing population density and the growth of social civilizations have brought about changing lifestyles. With the increase in industrial technologies, a vast urban waste problem has become the biggest paradox in the world [1,2,3,4,5,6].

Organic dyes are among the most dangerous pollutants causing water pollution. These dyes are frequently used in various industries, including the leather, plastic, food, paper, and textile industries [7,8,9,10].

Dyes are organic compounds that can give color to the environment in which they are present; they can be found in anionic, cationic, and non-ionic forms. Dyes with complex aromatic structures are stable, making them tough to eliminate from their present environment. Due to their aromatic properties, they also have a non-biodegradable design [11,12,13].

As mentioned, treating wastewater containing dyes, which are frequently used in many different industrial sectors, before discharge into the environment is very important for human health and the ecosystem. Even small amounts (less than 1 mg/L) can cause significant problems in terms of water pollution [14,15,16]. In particular, cationic dyes have harmful effects such as skin irritation and allergic reactions, as well as carcinogenic and mutagenic toxic effects [17].

In addition, the presence of industrial wastewater containing dyes discharged into the environment without adequate treatment prevents the photosynthesis process of aquatic plants by preventing the penetration of sunlight. This reduces the amount of dissolved oxygen and puts marine life under threat [18,19]. Today, more than 7 million tones of more than 100,000 types of dyes are produced [20].

Basic blue 3 (BB3) cationic dye, one of this study’s target dyes, is used in the textile industry’s carpet printing, wool coloring, and silk dyeing. However, when discharged without treatment, it negatively affects human health. For example, it can cause skin and eye damage, acute toxicity when exposed by inhalation, intestinal dysregulation, and cancer. These toxic effects occur more from paints with basic properties [21,22,23,24].

Another basic dye in this study is basic yellow 28 (BY28). It is one of the dyes frequently used in the dyeing of textile fibers. It is also used as an insecticide in aquarium environments. When mixed with water as waste, it has toxic properties that can harm aquatic animals and wildlife [25,26,27,28].

Paint wastewater can be treated using many different technological methods. For example, an electrochemical technique [29], a photochemical process [30], a microbial degradation method [31], treatment with ion exchange [32,33], a membrane separation method [34], and adsorption [35] are among the frequently used treatment methods. Among these, adsorption technology is one of the most preferred methods due to its low cost, high efficiency, and easy applicability [36,37].

Many different materials can be used as adsorbents. We can generally divide the materials used as adsorbents into two categories according to the source from which they are obtained. These can be listed as those obtained synthetically and those from natural sources.

If we want to give an example of synthetic adsorbents, β-cyclodextrin was prepared as an adsorbent with the use of a polypropylene-based substrate and a BB3 cationic dyestuff removal study was carried out [38]. In another study, nylon-6 nanofibers produced by using third-generation poly (propylene imine) dendrimers (PPI) were used as adsorbents and their ability to remove acid red 252 (AR252) dyestuff from aqueous media was studied [39]. In another study published in the literature, multicore cluster-based metal–organic frameworks (MOFs) showed remarkable efficacy in removing hazardous chemicals from wastewater due to their high porosity, functionality, and stability in water. In that context, a highly porous, water-stable Fe-MOF was produced. The efficiency of its acyl amide functional pores for organic dye removal was determined from the aqueous medium using methyl orange (MO), methyl red (MR), Congo red (CR), rose-bengal B (Ros B), rhodamine B (Rhod B), and methylene blue (MB), which have been successfully applied in the removal of different anionic and cationic dyes [40]. In another study, modifications were made to the MOF structures to increase the adsorption capacity of other dyes. These modifications included the addition of magnetic nanoparticles, other functional groups, and carbon-based nanomaterials such as graphene oxide and CNTs (carbon nanotubes). The results of removing different dyestuffs from aqueous media are comprehensively described [41].

On the other hand, natural adsorbents include activated carbon, sugar cane powder, defatted soya, corn cobs, microbial biomass, agricultural waste products, and coal [42,43,44,45,46,47,48]. However, alternative materials are being investigated for dye removal using economical, environmentally friendly, and abundant materials.

In the study presented, investigations were carried out on removing the toxic BB3 and BY28 dyes from aqueous solutions using plant powder obtained from the stem part of the Silybum marianum (milk thistle) plant, which has the abovementioned properties. Silybum marianum (SLM) is a plant that stands out with its pharmacological properties, especially with the extraction of its seed parts, in the literature. It is believed that its seeds, containing silymarin, have beneficial biological activities in addition to their antioxidant and anti-inflammatory effects [49,50,51,52].

The extracts obtained from these seeds are known to be used for therapeutic purposes in liver diseases, digestive system problems, upper respiratory tract infections, and lung inflammations, in addition to their dermatological benefits of reducing skin aging by their radical scavenging activity [53]. Moreover, they have alternative therapeutic properties for protecting cardiovascular health, being anti-diabetic, and clearing cancer indications. Therefore, they can help prevent the side effects of chemotherapy and radiation therapy [54,55,56,57].

Although there are many studies on using SLM seeds, there are no data on using the stem part of the mentioned plant as an adsorbent. Additionally, there are no studies on using the proposed adsorbent for treating wastewater containing the toxic dyes BB3 and BY28. The region where the SLM plant that was used was grown was Ashgabat city in Turkmenistan, a location that has not been previously studied for this data type.

In this study, experiments were first conducted to determine the protein, fatty acid, and carbohydrate contents, etc., to elucidate the structure of the SLM Stem part. Then, FTIR-ATR and SEM measurements were taken on SLM Stem-Natural powders exposed to different temperatures. Afterward, removal percentage experiments were conducted via treatment with aqueous solutions containing the BB3 and BY28 dyes. When the results of the optimization experiments were compared, SLM Stem-Natural and SLM Stem-800 °C were discovered to be the adsorbent materials with the best percentage removal. Under ideal circumstances, the removal procedures were repeated. However, this time the dye solutions were created with actual water samples rather than distilled water, and successful removal results were obtained again. Finally, the reusability of the adsorbent powders was investigated; it was found that the removal capacity continued successfully even after three consecutive regeneration processes.

## 2. Results and Discussion

### 2.1. Elucidation of the SLM Stem

Some analyses were carried out to elucidate the structure of the Silybum marianum stem. Firstly, the total protein amount of the Silybum marianum stem was obtained by working according to the Kjeldahl method [58]. Then, the amounts of polyunsaturated fatty acids, saturated fatty acids, monounsaturated fatty acids, and trans fatty acids were measured using gas chromatography (GC) according to the conditions given in the standard method [59]. The carbohydrate content was analyzed using the “method of determination by difference”. According to this method, the amounts of other components (protein, fat, etc.) in the structure of the analyzed sample at a certain weight were determined, the difference was taken, and the value found was expressed as carbohydrate content [60]. The total amount of fat was found by using the Soxhlet extraction method. Hexane was added to a given weight and taken into the Soxhlet apparatus. After applying the required procedure, the total fat amount was found from the weighing difference [61]. In the method used to find the amount of ash in the sample, oxidation was achieved at a controlled temperature in an electric furnace and the total ash content was found from the weighing difference [62]. The moisture content was determined from the weighing difference obtained by applying the relevant procedure to the sample at a certain weight in an oven heated to 70 °C [63]. All results obtained are given in Table 1.

The stem structure, generally used as waste or animal feed, contains 13.9 g/100 g protein. In addition, it was observed to have a rich composition by containing various fatty acid components and to be like essential nutrients that provide energy, with a quantity of 68.93 g/100 g of carbohydrates.

### 2.2. Characterization of the Adsorbent Obtained from SLM Stem

**Figure 1:**
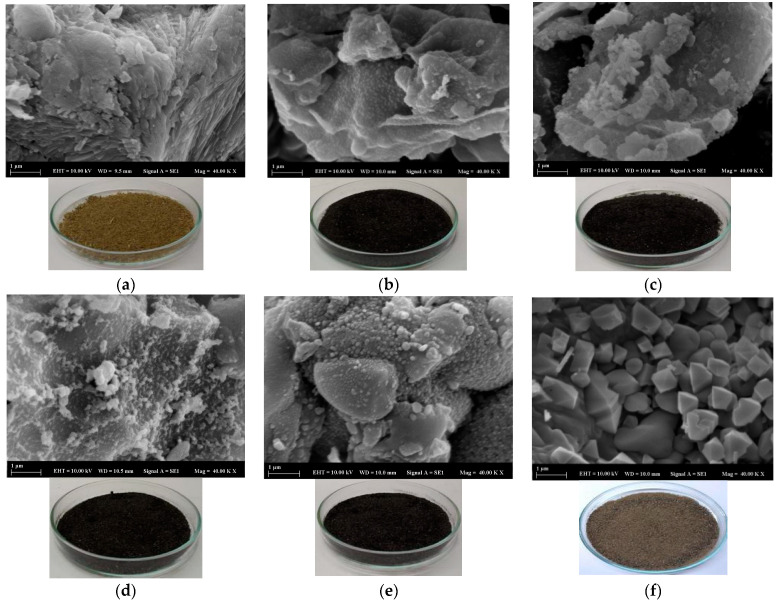
SEM images and photos of the SLM Stem powder after different carbonized temperatures: SLM Stem-Natural 50 °C (**a**), 200 °C (**b**), 400 °C (**c**), 600 °C (**d**), 800 °C (**e**), and 900 °C (**f**).

SEM analyses can help reveal surface characteristics. The SEM images in Figure 1 show images of adsorbent materials carbonized at temperatures of 200 °C (b), 400 °C (c), 600 °C (d), 800 °C (e), and 900 °C (f), as well as the SLM Stem-Natural powder SEM image (a), which has not been subjected to any calcination process. The SEM image of the SLM Stem-Natural powder presents a porous structure suitable for adsorption, whereas as the calcination temperature increases, a flatter pore structure is observed. However, at 800 °C, these ridged structures increase again, resulting in a higher adsorption percentage. On the other hand, more cubic crystalline surfaces are observed at 900 °C than porous structures. This structure is also supported by the fact that less colorant removal was obtained when this adsorbent was used during the experimental studies.

**Figure 2:**
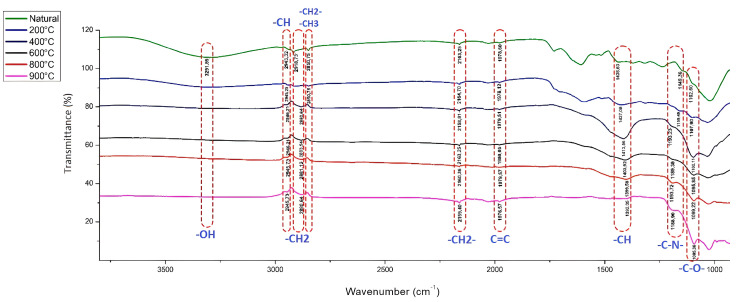
The FTIR−ATR spectra of SLM Stem-Natural and SLM Stem powder after different carbonized temperatures between 200 and 900 °C.

As shown in Figure 2, the FTIR-ATR spectra of the plant powders exhibited significant changes in the intensity and position of several peaks as the carbonized temperature was increased. The broad peak observed at 3291 cm^−1^ in the natural sample was absent in all the subsequent examples, indicating a loss of the associated functional group. The band present at 3291 cm^−1^ was related to the pH_zpc_ 5.4 value of SLM Stem-Natural, whereas it was not seen in SLM Stem-800 °C. The pH_zpc_ value of SLM Stem-800 °C was found to be 9.6; it is thought that the structure found in this way can be supported. The weak and broad peak between 2945 and 2943 cm^−1^, which can be attributed to C-H stretching vibrations, was observed in all the samples. Similarly, the overall peak between 2883 and 2880 cm^−1^, corresponding to CH_2_ asymmetric stretching vibrations, was absent in the first two samples but was observed in the last four samples, indicating a shift in functional group composition with increasing decarburization temperature.

The appearance of the SLM Stem-Natural (Figure 1a) is a light brown-yellow mixture, whereas the SEM images show the formation of indented layers. However, when a temperature of 200 °C is applied, as seen in Figure 1b, the plant powders turn black and the carbonization process starts. Notably, the layers seen in the SEM images become less rough. When the temperature applications are increased up to 900 °C and the FTIR-ATR spectra are analyzed, it can be stated that the removal of the volatile parts from the environment leads to the more precise observation of structural elements, such as aliphatic parts (-CH_2_ groups), and the surfaces turn into more clearly defined structures, as shown in Figure 1f.

The weak and relatively sharp peak observed at 2850 cm^−1^ in the first two samples, which can be attributed to the C-H stretching vibrations of methylene groups, was absent in the last four samples, suggesting a loss of the associated functional group. Additionally, the peak observed at 2850 cm^−1^ in the previous four samples was inverted, indicating changes in the stereochemistry of the methylene group.

The sharp but weak peak observed between 2164 and 2158 cm^−1^, which can be attributed to the C-H bending vibrations of methylene groups, showed a decrease in intensity at 800 °C but increased in intensity at higher carbonized temperatures. The weak sharp peak observed between 1980 and 1976 cm^−1^, which can be attributed to C=C stretching vibrations, also showed a similar trend, with a decrease in intensity at 800 °C and an increase in intensity at 900 °C.

The broad and robust peak observed between 1438 and 1392 cm^−1^, which corresponds to the C-H bending vibrations of aliphatic groups, was observed in all samples. However, its intensity was significantly higher in the example carbonized at 400 °C. The broad and weak peak observed between 1193 and 1149 cm^−1^, which can be attributed to C-N stretching vibrations, was observed in all the samples, but its intensity was higher in the pieces carbonized at 800 °C and 900 °C. Similarly, the broad and medium peak observed between 1107 and 1095 cm^−1^, corresponding to C-O stretching vibrations, was observed in all samples but had weaker intensity in the natural selection. Overall, the FT-IR spectra of the plant powders showed significant changes in the functional group composition with increasing carbonized temperature, indicating this parameter’s importance in preparing plant-based materials for various applications.

**Figure 3:**
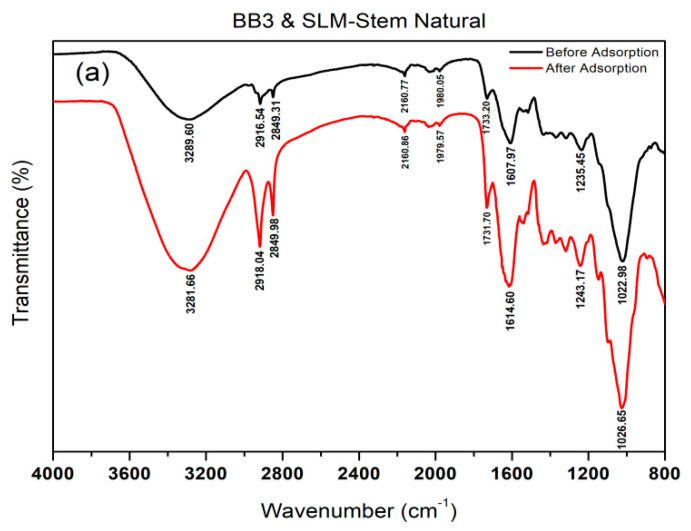

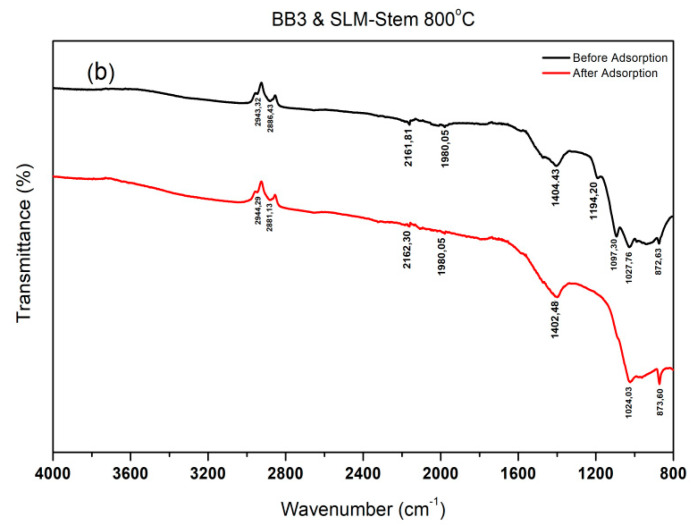
The before and after FTIR−ATR spectra of BB3 adsorption on SLM Stem-Natural (**a**) and SLM Stem-800 °C (**b**).

As shown in Figure 3a, the FTIR-ATR spectra comparison of the SLM Stem-Natural before and after the BB3 dye adsorption process showed that the peak at 3289 cm^−1^ remained unchanged, whereas the peaks at 2916 cm^−1^ and 2850 cm^−1^ became more intense in the post-adsorption spectrum. Furthermore, the peaks at 2160 cm^−1^, 2029 cm^−1^, 1979 cm^−1^, and 1609 cm^−1^ were unchanged, whereas the peak at 1732 cm^−1^ became sharper and more intense in the post-adsorption spectrum. Additionally, the peaks at 1436 cm^−1^ and 1154 cm^−1^ were more robust and sharper in the post-adsorption spectrum compared with the pre-adsorption spectrum.

In Figure 3b, the FTIR-ATR spectra of the SLM Stem-800 °C before and after the BB3 dye adsorption process revealed that the peak at 1194 cm^−1^ was absent in the post-adsorption spectrum. In contrast, the peaks at 1402–1404 cm^−1^ and 1024–1027 cm^−1^ were preserved. Additionally, a peak at 873 cm^−1^ was observed to be stronger in the post-adsorption spectrum compared with the pre-adsorption spectrum.

**Figure 4:**
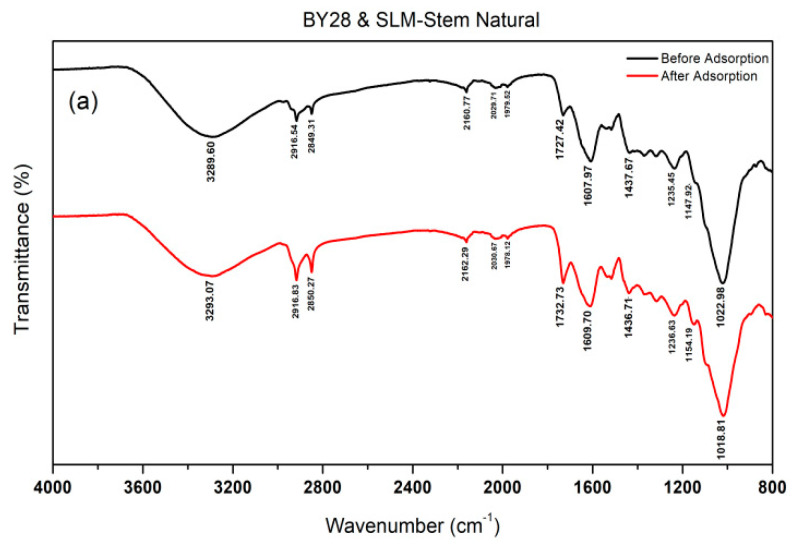

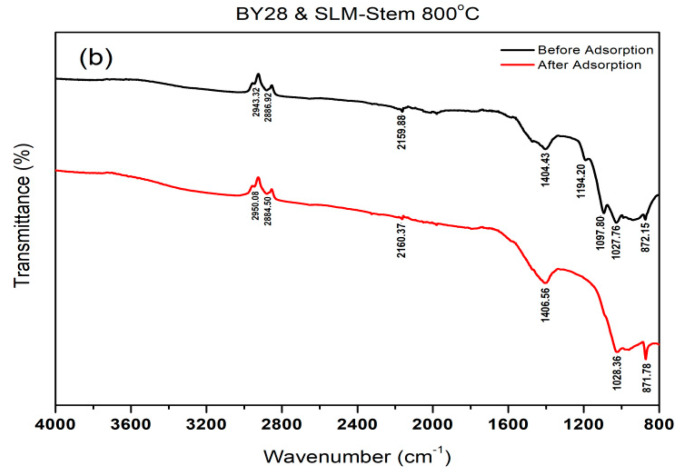
The FTIR−ATR spectra of before and after adsorption of BY28 on SLM Stem-Natural (**a**) and SLM Stem-800 °C (**b**).

In Figure 4a, the comparison of the FTIR-ATR spectra of the SLM Stem-Natural before and after the BY28 dye adsorption process indicated that the peak at 3291 cm^−1^ remained unchanged. In contrast, the peaks at 2944 cm^−1^ and 2851 cm^−1^ were more intense in the post-adsorption spectrum compared with the pre-adsorption spectrum. The peaks at 2162 cm^−1^, 2131 cm^−1^, 1456 cm^−1^, and 1026 cm^−1^ were preserved in both spectra. Moreover, the peak at 1740 cm^−1^ was sharper and more intense in the post-adsorption spectrum compared with the pre-adsorption spectrum, whereas the peak at 1243 cm^−1^ was stronger in the post-adsorption spectrum compared with the pre-adsorption spectrum.

The FTIR-ATR spectra of the SLM Stem-800 °C (Figure 4b) before and after the BY28 dye adsorption process revealed several changes. The peaks at 2943 and 2886 cm^−1^ remained unchanged, whereas the peaks at 2916 cm^−1^ and 2849 cm^−1^ became more intense. The 2159 cm^−1^ and 1404 cm^−1^ peaks were unchanged, whereas those at 1194 cm^−1^ and 1097 cm^−1^ disappeared. Furthermore, the peak at 1027 cm^−1^ became broader and less intense and the peak at 872 cm^−1^ became more intense. These changes in the FTIR-ATR spectra indicate alterations in the adsorbent’s functional groups and molecular vibrations, possibly due to the adsorption process.

**Figure 5:**
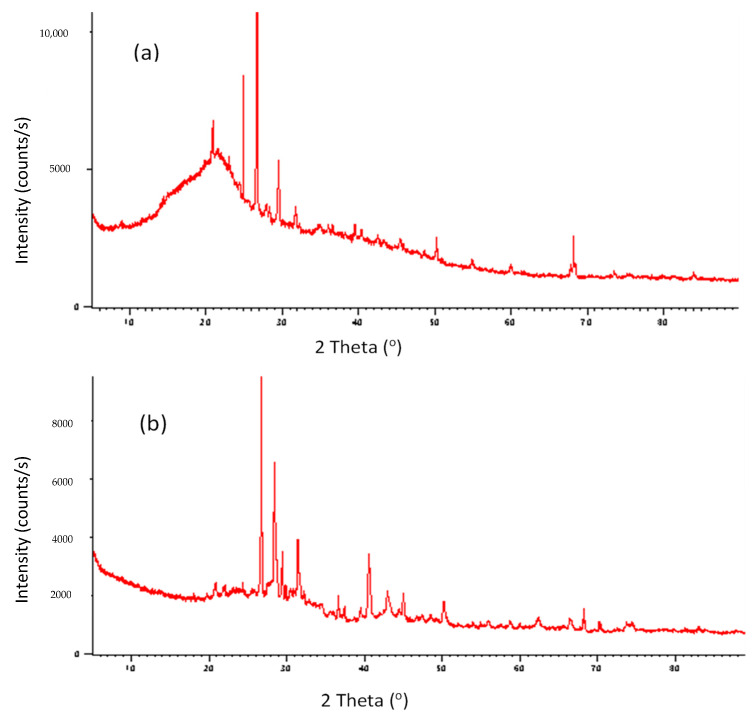
The XRD pattern of SLM Stem-Natural (**a**) and SLM Stem-800 °C (**b**).

As observed from the XRD graph, the structure in Figure 5a represents the data for the SLM Stem-Natural plant powder. In Figure 5b, the XRD measurements of the crystal structure values obtained by exposing the SLM Stem-800 °C are expressed. The diffraction peaks in the structure before the heating process are at 2θ of 20.92°, 24.91°, 26.68°, 29.53°, 31.76°, 39.54°, 50.26°, and 68.22°. When the values of the structure after being exposed to 800 °C are examined, a noticeable increase in sharp peaks, where the crystalline structure is more supported, can be observed. The diffraction peaks are listed as 2θ at 20.89°, 24.35°, 26.72°, 28.40°, 31.42°, 40.56°, 42.95°, 45.00°, 50.16°, and 68.19°.

**Figure 6:**
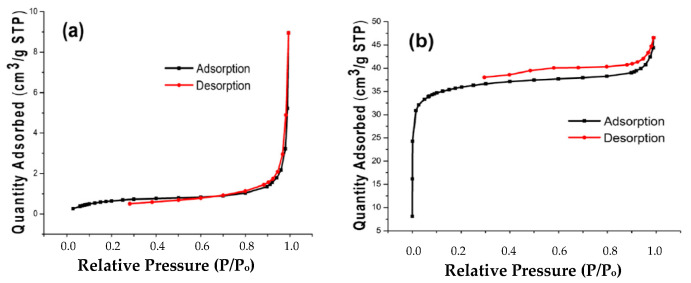
The BET pattern of SLM Stem-Natural (**a**) and SLM Stem-800 °C (**b**).

Figure 6a,b show the nitrogen adsorption–desorption isotherms of SLM Stem-Natural and SLM Stem-800 °C, respectively. Based on Figure 6, it can be seen that a type II isotherm is shown in (a) and type I isotherm is shown in (b). The SLM Stem-Natural’s surface area, pore volume, and half-pore width were found to be 2.648 m^2^/g, 0.014 cm^3^/g, and 20.898 nm, respectively, based on the findings of the BET study. Similar to this, the SLM Stem-800 °C’s surface area, pore volume, and half pore width were found to be 122.564 m^2^/g, 0.072 cm^3^/g, and 2.350 nm, respectively. Adsorbents are typically categorized based on their average pore diameter into macroporous (>50.0 nm), mesoporous (2.0–50.0 nm), microporous (0.7–2.0 nm), and ultra-microporous (<0.7 nm) [64,65]. In this case, it was determined that the SLM Stem-Natural is mainly a non-porous material and that the SLM Stem-800 °C has a microporous structure.

The zeta potential values of SLM Stem-Natural and SLM Stem-800 °C were −36.6 mV and −33.36 mV, respectively.

### 2.3. Determination of pH Zero-Point Charge

**Figure 7:**
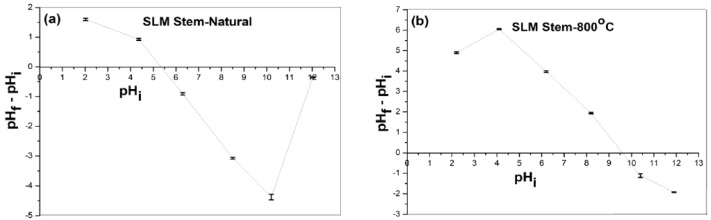
The values of pH_zpc_ of SLM Stem-Natural (**a**) and SLM Stem-800 °C (**b**).

The pH zero-point charge (pH_zpc_), one of the key variables influencing the adsorption procedure is crucial. The pH_zpc_ values of the SLM Stem-Natural and SLM Stem-800 °C adsorbents were found using the drift method [66,67]. Accordingly, six 50 mL bottles were filled with solutions with initial pH (pHi) values ranging from 2 to 12 and 0.50 g of SLM Stem-Natural adsorbent was added. The mixtures were kept at room temperature for 24 h. Then, the solutions’ final pH values (pHf) were subtracted from the initial pH values and the ΔpH values were calculated. A graph was created by writing pHi on the X axis and ΔpH (pHf–pHi) on the Y axis. The same procedure was repeated using SLM Stem-800 °C as an adsorbent. The point ΔpH = 0 indicates the pHpzc value of the adsorbent used. Accordingly, as seen in Figure 7a,b, the pH_zpc_ values of SLM Stem-Natural and SLM Stem-800 °C were found to be 5.4 and 9.6, respectively.

### 2.4. Determination of the Active Groups of the Adsorbent

To identify the presence of acidic and basic groups on the SLM Stem-Natural and SLM Stem-800 °C, titration tests were used as described in the literature. A 0.1 N NaOH solution neutralized the total acid sites, matching the carboxylic, phenolic, and lactonic sites [68]. In contrast, a 0.1 N HCl solution neutralized the basic sites. For this purpose, 100 mL of titrating solution was taken into the volumetric flask and then 5 g of SLM Stem-Natural was added to it. The flask was slowly stirred and partially submerged in a water bath with a constant temperature of 25 °C for five days. Then, a 2 mL sample was titrated with a 0.1 N HCl or NaOH solution. All titrations were repeated three times. The titration was carried out using the WTW Series pH 720 device [69]. The same procedure was applied again using SLM Stem-800 °C as an adsorbent.

In conclusion, the concentration of the total acidic and basic groups on the surfaces of SLM Stem-Natural and SLM Stem-800 °C are shown in Table 2. The acidic and basic groups were determined from the amphoteric properties of components such as proteins and amino acids in the plant structure. However, due to the excess concentration of basic groups, it was concluded that the SLM Stem-Natural and SLM Stem-800 °C surfaces have a higher basic character. If we want to compare the values of both adsorbents with each other, it can be thought that the basic nature of SLM Stem-800 °C will be greater than that of SLM Stem-Natural. When the pH effect is examined, it can be considered that the presence of increasing negative charges in SLM Stem-Natural at pH > pHzpc = 5.4 (for BY28 optimum pH 6, R% 62; for BB3 optimum pH 7, R% 86.79) and SLM Stem-800 °C at pH > pHzpc = 9.6 (for BY28 optimum pH 10, R% 93.45; for BB3 optimum pH 10, R% 97.95) mean that the optimum pH values in this area may support the increasing basic character. SLM Stem-800 °C’s higher percentage of dyestuff removal than SLM Stem-Natural may result from its stronger basic nature.

### 2.5. The Impact of Adsorbent Amount on the Adsorption of BB3 and BY28

The variation in adsorbent amount is one of the most important factors influencing the adsorption process and the removal percentage value. The removal experiments for the BY28 and BB3 dyes were investigated using SLM Stem-Natural and carbonized SLM-Stem plant powders varying from 200 °C to 900 °C. The other parameters were kept constant at C_0_ = 30 mg/L, V= 10 mL, pH ≅ 6.5, 200 rpm, 21.3 °C, and 30 min. The adsorbent amount was varied between 0.01 g and 0.2 g and experiments were repeated.

**Figure 8:**
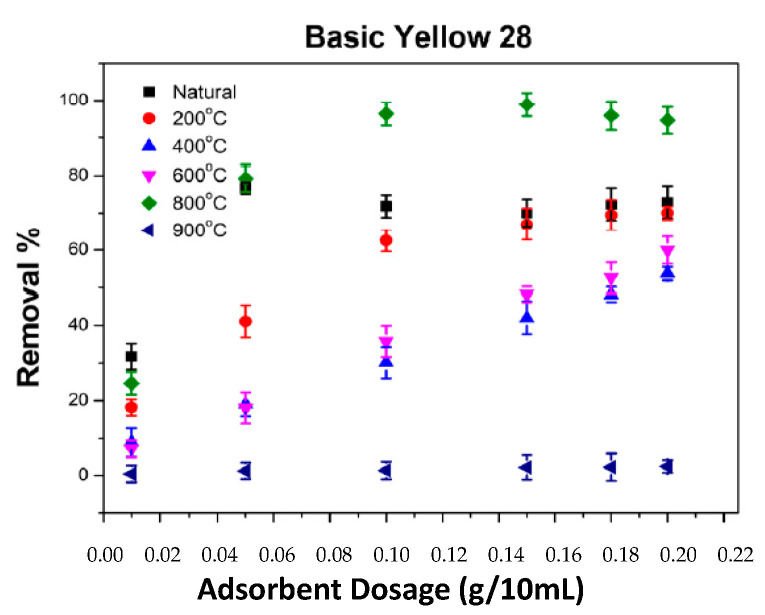
The impact of various ash temperatures on the adsorption of BY28 dye on SLM Stem.

**Figure 9:**
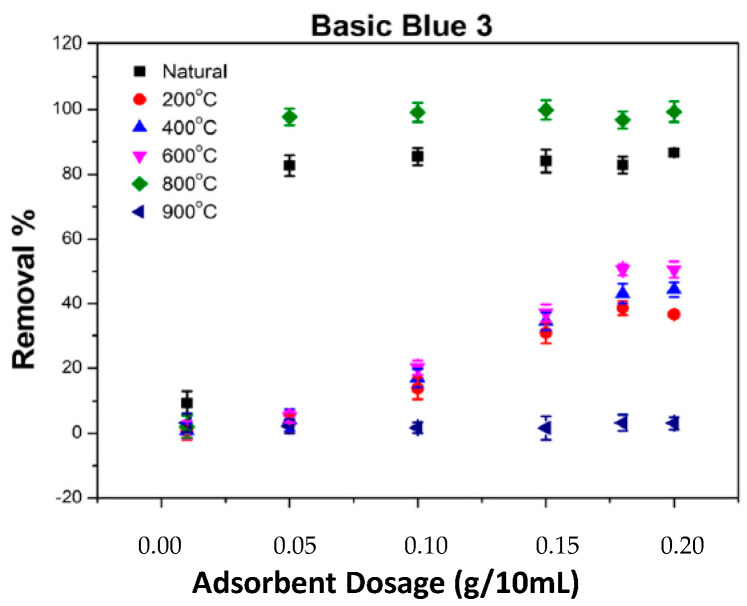
The impact of various ash temperatures on the adsorption of BB3 dye on SLM Stem.

When the data in Figure 8 and Figure 9 were examined, it was determined that SLM Stem-Natural plant powder and SLM Stem-800 °C had the highest percentage removal values for the toxic dyes BY28 and BB3. Therefore, optimization data for these two adsorbents were emphasized in ongoing studies.

**Figure 10:**
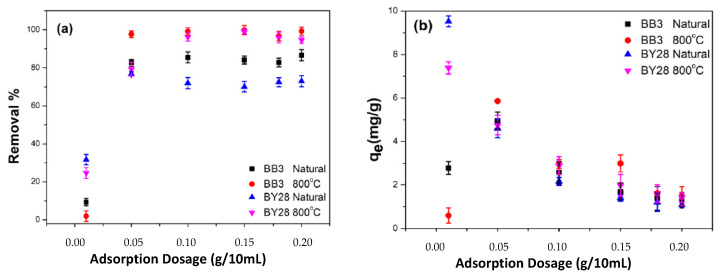
The effects of various adsorbents dosages on the adsorption of BB3 and BY28 dyes on SLM Stem-Natural and SLM Stem-800 °C. Removal % (**a**), q_e_ (**b**).

Keeping the abovementioned parameters constant, the removal percentage and q_e_ values of BY28 dye were calculated as shown in Figure 10a,b. Accordingly, it was observed that the best removal percentage achieved was 74% using 0.05 g of SLM Stem-Natural as an adsorbent in the study. In the ongoing study under the same conditions, particles of SLM Stem-800 °C were used as adsorbent and an optimum removal percentage of 98.90% was achieved with the addition of 0.15 g of adsorbent.

On the other hand, in the repeated experiments with BB3 dye, the percentage removal values obtained from the studies using SLM Stem-Natural and SLM Stem-800 °C as adsorbents, were 86.62% and 99.82%, respectively, as shown in Figure 10a. Additionally, all calculated q_e_ values are shown in Figure 10b. The optimum adsorbent amounts were SLM Stem-Natural 0.2 g and SLM Stem-800 °C 0.15 g.

### 2.6. The Effect of the Initial Solution Concentration on BB3 and BY28 Adsorption

Another parameter affecting the removal value is the initial concentration of the studied dyestuff at varying concentrations ranging from 10 to 50 mg/L. In the experiments using SLM Stem-Natural as the adsorbent for BY28, the adsorbent amount was kept at 0.05 g, whereas the other parameters were fixed at V = 10 mL, pH ≅ 6.5, 200 rpm, 21.3 °C, and 30 min. The best removal percentage obtained was 78.26% for an initial 15 mg/L concentration. The study was continued using the SLM Stem-800 °C particles as the adsorbent, and the BY28 removal experiments were repeated using the optimum adsorbent amount of 0.15 g. The result showed a removal percentage of 98.77% for an initial 30 mg/L concentration.

**Figure 11:**
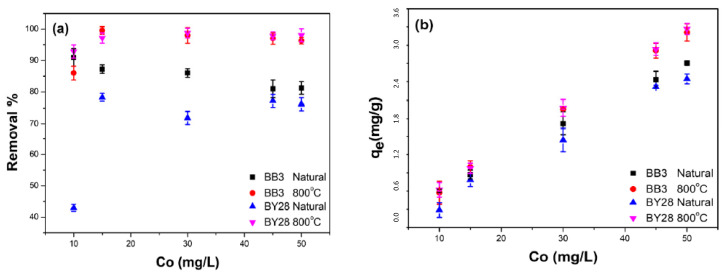
The influence of initial concentration on the adsorption of BB3 and BY28 dyes on SLM Stem-Natural and SLM Stem-800 °C. Removal % (**a**), q_e_ (**b**).

The same experiments were repeated with BB3, but this time the adsorbent dosage was 0.2 g for SLM Stem-Natural and 0.15 g for the particles of SLM Stem-800 °C. The removal percentage was found to be 90.97% for the initial concentration of 10 mg/L and 99.58% for the initial concentration of 15 mg/L, respectively. All the removal percentages and q_e_ values are shown in Figure 11a,b.

### 2.7. Effect of Contact Time on BB3 and BY28 Adsorption

**Figure 12:**
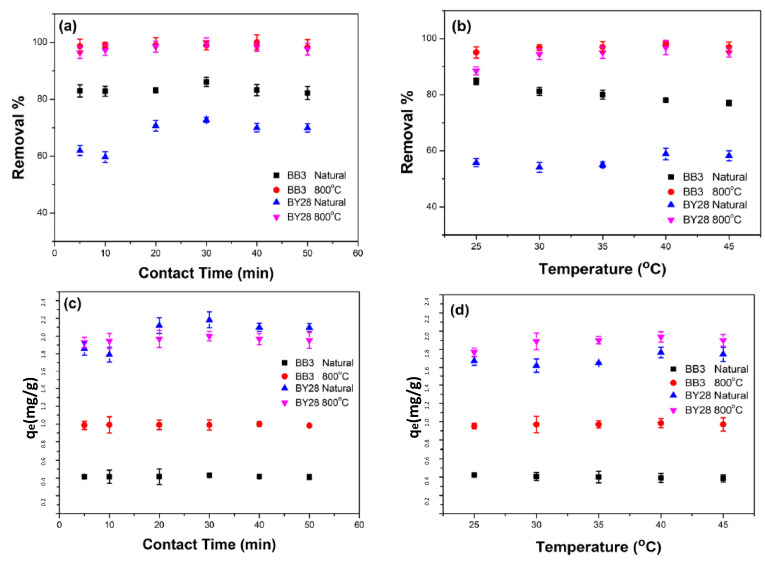
The influence of contact time ((**a**): Removal %, (**c**): q_e_) and temperature ((**b**): Removal %, (**d**): q_e_) on the adsorption of BB3 and BY28 dyes on SLM Stem-Natural and SLM Stem-800 °C.

A crucial factor in determining adsorption effectiveness is contact time. Various contact times between 5 and 50 min were used to measure the impact of contact time on the adsorption process, whereas the other parameters were kept constant: V = 10 mL, pH ≅ 6.5, and 200 rpm. When working with 0.05 g of SLM Stem-Natural and an initial concentration of 15 mg/L for BY28 dye, previously determined parameters, the results were found to be 72.75% and q_e_ = 2.18 at 30 min. When working with 0.15 g SLM Stem-800 °C and an initial concentration of 30 mg/L, the results were found to be 99.99% and q_e_ = 1.99 at 30 min. For BB3 dye, with previously determined parameters of 0.2 g SLM Stem-Natural and an initial concentration of 10 mg/L, the results obtained were 86.09% and q_e_ = 0.43 at 30 min. When working with 0.15 g SLM Stem-800 °C and an initial concentration of 15 mg/L, the results were 99.99% and q_e_ = 0.99 at 40 min. All obtained values are shown in Figure 12a,c.

### 2.8. The Effect of Ambient Temperature on the Adsorption of BB3 and BY28

Ambient temperature is another factor that impacts the adsorption procedure and effectiveness. Keeping parameters such as pH 6.5 and 200 rpm constant, at different temperatures ranging from 25 to 40 °C, the impact of ambient temperature on the adsorption process was evaluated. When SLM Stem-Natural was used for the BY28 dye, the previously determined optimal values of 0.05 g of adsorbent, 15 mg/L initial concentration, and 30 min of contact time were achieved. According to the results, the best removal percentage was 58.84% and q_e_ = 1.77 was reached at 40 °C when 0.15 g of SLM Stem-800 °C was used as the adsorbent with a 30 mg/L initial concentration and 30 min of contact time. The results showed a removal percentage of 96.86% and q_e_ = 1.94 at 40 °C.

For BB3 dye, the best removal efficiency was obtained at 25 °C, with a removal percentage of 84.71% and q_e_ = 0.42 when SLM Stem-Natural was used with the previously determined parameters of 0.2 g adsorbent, 10 mg/L initial concentration, and 30 min of contact time. When the experiments were conducted using 0.15 g of SLM Stem-800 °C with a 15 mg/L initial concentration and 40 min of contact time at 40 °C, the removal percentage was found to be 98.32% and q_e_ = 0.98. The experimental removal percentages are shown in Figure 12b,d.

### 2.9. Effect of pH on BB3 and BY28 Adsorption

The initial pH plays a vital role in evaluating the adsorption process and removal percentage by controlling the amount of ionization of the dye molecule and the adsorbent’s surface charge; using dye solutions with various pH ranges of 2 to 12 allowed for evaluating the initial pH influence on the removal percentage. The BY28 dye was used under the same conditions of 0.05 g of SLM Stem-Natural, 15 mg/L starting concentration, 30 min of contact time, and 40 °C. The best initial pH was 6, with a removal percentage of 62%. When 0.15 g of SLM Stem-800 °C was used with a 30 mg/L initial concentration, 30 min of contact time, and a 40 °C temperature, the best result was obtained at pH 10, with a removal percentage of 93.45%.

**Figure 13:**
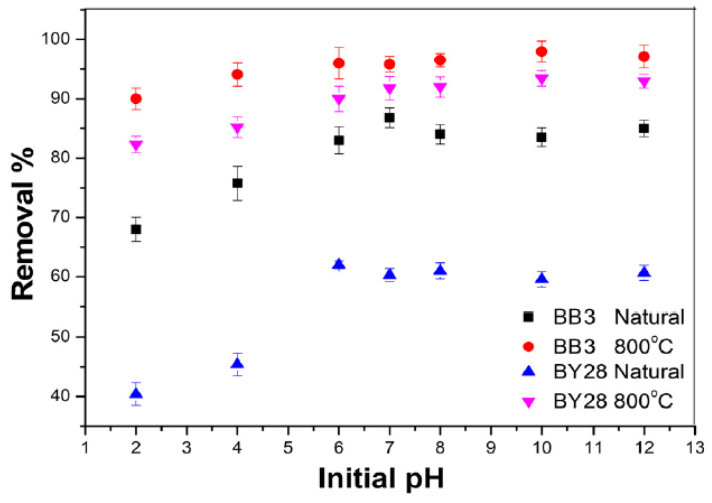
The influence of initial pH on BB3 and BY28 dye adsorption on SLM Stem-Natural and SLM Stem-800 °C.

For BB3 dye, an amount of 0.2 g of SLM Stem-Natural, an initial concentration of 10 mg/L, 30 min of contact time, and a temperature of 25 °C were kept constant. The best initial pH was 7, with a removal percentage of 86.79%. In the continuing study, 0.15 g of SLM Stem-800 °C was used with a 15 mg/L initial concentration, 40 min of contact time, and a temperature of 40 °C. The best initial pH was 10, with a removal percentage of 97.95%. All removal percentage values obtained are presented in Figure 13.

To have information about the electrical charge interaction between the surfaces during adsorption, evaluating the optimum pH values in pH_zpc_ is essential [67]. At pH < pH_pzc_, the surface charges of the sorbent are positive and sensitive to electrostatic interactions with anionic dye molecules. If pH > pH_pzc_, the surface of the adsorbent becomes negatively charged and can be used appropriately for the adsorption of cationic dyes such as BB3 and BY28 [66,70]. The pH_pzc_ values of the adsorbents were found to be 5.4 and 9.6 for SLM Stem-Natural and SLM Stem-800 °C, respectively (Figure 7a,b). As shown in Figure 13, where the effect of pH is evaluated, optimum BB3 and BY28 cationic dye removal could not be achieved below these values. This is because when pH < pH_pzc_, the surface charges of the sorbent are favorable and the number of negatively charged sorption sites to which the cationic dye can bind electrostatically decreases. This leads to a decrease in the amount of cationic dye that can be adsorbed and a low percentage removal value.

As seen in Figure 7a, the SLM Stem-Natural pH_pzc_ is 5.4. This is supported by the optimum values, pH = 7 for BB 3 and pH = 6 for BY28, which are found to increase the percentage removal values when the pH value of the medium is >5.4. Again, as seen in Figure 7b, the SLM Stem-800 °C pH_pzc_ is 9.6. The optimum values found when the pH value of the medium increases to over 9.6 are compatible with the pH_pzc_ values determined experimentally, which are pH 10 for BB3 and pH 10 for BY28.

### 2.10. The Effect of Agitation Speed on BB3 and BY28 Adsorption

Agitation speed also plays an essential role in the adsorption process and the efficiency of it. The effect of agitation speed was evaluated with rates ranging from 100 to 200 rpm. For the BY28 dye, 0.05 g of SLM Stem-Natural, an initial concentration of 15 mg/L, a contact time of 30 min, and a temperature of 40 °C were kept constant at pH 6. The best result was obtained at 100 rpm, with a removal efficiency of 58.12%. When working with 0.15 g of SLM Stem-800 °C, the best result was obtained at 150 rpm, with a removal percentage of 98% under the conditions of an initial concentration of 30 mg/L, a contact time of 30 min, a temperature of 40 °C, and a pH 10.

**Figure 14:**
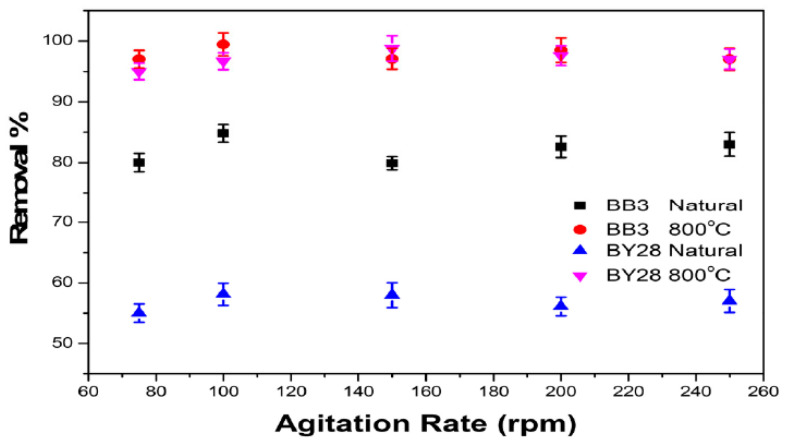
The effect of agitation rate on BB3 and BY28 dye adsorption on SLM stem-Natural and SLM Stem-800 °C.

For the BB3 dye, an amount of 0.2 g of SLM Stem-Natural, an initial concentration of 10 mg/L, a contact time of 30 min, and a temperature of 25 °C were kept constant at pH 7. The best result was obtained at 100 rpm, with a removal percentage of 84.82%. In the ongoing study, when experiments were repeated with 0.15 g of SLM Stem-800 °C under the conditions of an initial concentration of 15 mg/L, a contact time of 40 min, a temperature of 40 °C, and a pH of 10, the highest removal percentage of 99.45% was observed at 100 rpm. All obtained removal efficiency values are presented in Figure 14.

The removal percentage values in Figure 14 climb until a particular point and then decline again. With increased agitation speed, the adsorbent materials are distributed in the dye solution and the surfaces that the dye molecules can achieve are suitable for adsorption. Nevertheless, speeding up the agitation may lower the R% values by degrading the stable film surface and adversely influencing it. Studies in the literature have also shown comparable outcomes [71]. The R% numbers shown above were attained in this study.

### 2.11. Real Wastewater Experiments

**Figure 15:**
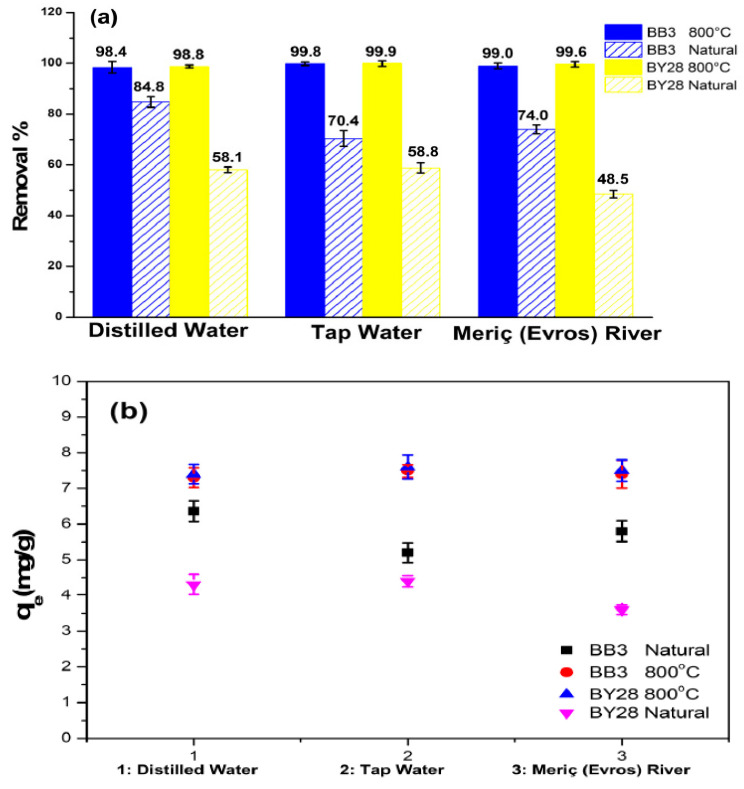
The real sample application. (**a**): Removal %, (**b**): q_e_.

The removal efficiencies of the adsorbent types obtained from SLM Stem-Natural and SLM Stem-800 °C were repeated using actual water samples. For this purpose, wastewater samples containing BB3 and BY28 dyes were prepared using tap water, Meriç (Evros) River flowing water, and distilled water. All experiments used 15 mg/L, 10 mL dye solution, and 0.01 g adsorbent materials. The data obtained are shown in Figure 15a,b. According to these results, it is observed that the most successful BY28 dye removal percentage and q_e_ value were achieved with 99.9%, q_e_ = 7.49 and 99.6%, q_e_ = 7.47 when the SLM Stem-800 °C adsorbent was used in studies containing tap water and Meriç (Evros) River water, respectively. In the studies containing BB3 dye, a successful treatment process was achieved again using SLM Stem-800 °C in the solutions containing tap water and Meriç (Evros) River water, with removal percentages and q_e_ values found to be 99.8%, q_e_ = 7.48 and 99%, q_e_ = 7.42, respectively. According to the data obtained, it seems that wastewater treatment studies give good results using SLM Stem-Natural, especially SLM Stem-800 °C adsorbents.

### 2.12. Reusability

**Figure 16:**
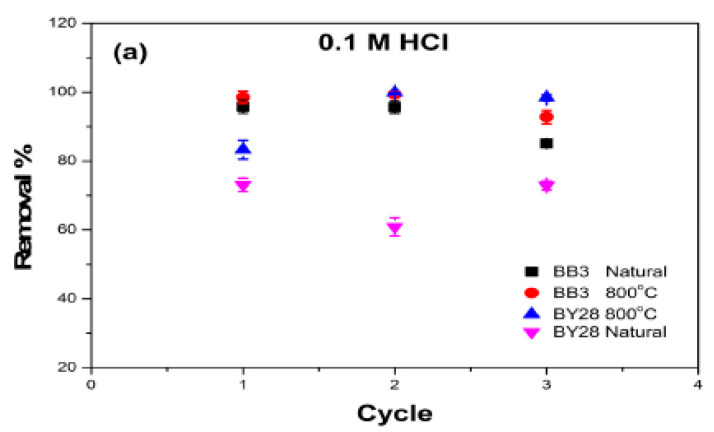

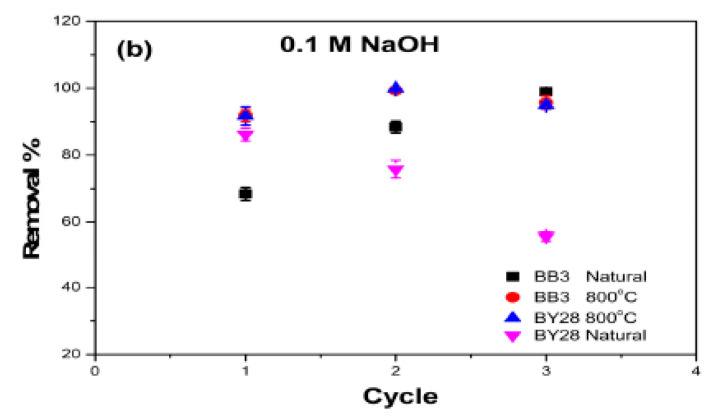
The reusability behavior of SLM Stem-Natural and SLM Stem-800 °C: (**a**) 0.1 M HCl and (**b**) 0.1 M NaOH.

Reusability tests for the SLM Stem-Natural and SLM Stem-800 °C adsorbents were separately examined for both dyes using the batch method applied during all experiments. The desorption of adsorbent surfaces was studied separately using 0.1 M HCl and 0.1 M NaOH solutions. The results are shown in Figure 14. After three repetitions, the SLM Stem-800 °C showed an excellent reusability of 98.4% in BY28 removal studies carried out with 0.1 M HCl. It was also found that the SLM Stem-800 °C was successfully used in BB3, with a removal percentage of 99% after three repetitions in 0.1 M NaOH regeneration. Furthermore, as seen in Figure 16, the other removal percentage values also contain significantly high reusability. In conclusion, the reusability of the adsorbents obtained from SLM-stem powders shows successful results in purifying the expressed dyes from wastewater.

### 2.13. Adsorption Isotherms

Adsorption isotherms can provide valuable insights into the binding mechanisms of dyes to the utilized adsorbents, as well as information about the adsorption capacity and affinity. In this study, Langmuir and Freundlich isotherm models were employed to investigate the adsorption behavior of the BB3 and BY28 dyes using the SLM Stem-Natural and SLM Stem-800 °C adsorbents.

**Figure 17:**
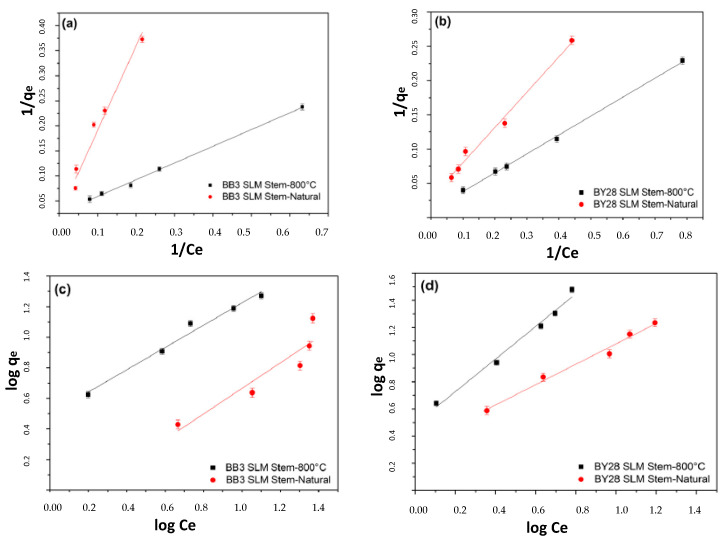
Langmuir (**a**,**b**) and Freundlich (**c**,**d**) adsorption isotherm plots for the adsorption of BB3 and BY28 onto SLM Stem-Natural and SLM Stem-800 °C (pH: 6, 30 mg/L, V: 50 mL, m: 0.1 g).

As shown in Figure 17a,b, Langmuir isotherm curves were generated from the experimental data. The adsorption equilibrium constant K_L_ and maximum adsorption capacity q_max_ were calculated from the graphs plotted between 1/q_e_ and 1/C_e_. According to the values presented in Table 3, Langmuir’s behavior, indicating monolayer adsorption, was observed.

Figure 17c,d display the straight lines representing the Freundlich isotherm equation obtained from the graphs plotted between log q_e_ and log C_e_. The Freundlich constant “1/n” determines the adsorption intensity and all values presented in Table 3 are less than 1, indicating physical adsorption. In this study, the Freundlich adsorption capacity “K_f_” increases with larger values, ranging from 0.7335 to 3.4516.

Examining the results in Table 3, the linear regression coefficient “r^2^” for the Langmuir isotherm varies between 0.9987 and 0.9720, whereas for the Freundlich isotherm, it ranges from 0.9863 to 0.8593, confirming monolayer adsorption. When using SLM Stem-Natural as the adsorbent, the maximum adsorption capacities for the BB3 and BY28 dyes were found to be 13.9645 and 36.5497 mg/g, respectively, whereas using SLM Stem-800 °C as the adsorbent resulted in values of 36.8053 and 271.7391 mg/g, respectively. This indicates the highest capacity for removing BY28 dye was achieved using SLM Stem-800 °C as the adsorbent.

### 2.14. Comparative Research

The maximum adsorption capacities of different adsorbents used to remove the BB3 and BY28 dyes from aqueous solutions are compared in Table 4. As shown, the adsorption capacity values obtained for BB3 and BY28, mainly when using the SLM Stem-800 °C adsorbent, indicate that it has the highest adsorption capacity after the study using “Smectite-rich natural clays (Ghassoul)” [25]. This demonstrates the potential of the adsorbents used in the study as promising candidates for successfully removing both BB3 and BY28 dyes from aqueous solutions considering their natural origin, low cost, and non-negligible adsorption capacities against these toxic dyes. Thus, they can be used as potential adsorbents in future studies to remove these poisonous dyes from aqueous solutions.

## 3. Experimental

### 3.1. Materials and Methods

Silybum marianum (milk thistle) is an annual or biennial plant belonging to the Asteraceae family. The length of its fruits can be 15–20 mm, whereas the length of its leaves can be 25–50 cm and their width can be 12–25 cm. The stem of the plant can vary from 20 cm to 150 cm. Although it is native to North America and Asia, it can also be found in Southern Europe, the Russian Federation, South America, Australia, China, Central Europe, and Pakistan [69]. As a wild and invasive plant commonly found in Turkey, Silybum marianum was collected from the city of Ashgabat in Turkmenistan for the experiments in this study. The cationic dyes used in the experiments, basic blue 3 (MW = 359.89 g/mol, λ_max_ = 654 nm C_20_H_26_ClN_3_O) and basic yellow 28 (MW = 433.5 g/mol, C_21_H_27_N_3_O_5_S, λ_max_ = 438 nm), were obtained from Sigma-Aldrich. The 0.1 M HCl and 0.1 M NaOH solutions used in the experiments were prepared by Merck. The pH changes were achieved using buffer solutions at the appropriate pH values. All necessary pH measurements during the regeneration experiments were carried out using a WTW Series pH 720 device. A Julabo SW 22 heated shaking water bath was used throughout the batch adsorption experiments. Distilled water was obtained from the Milli-Q (Millipore, Burlington, MA, USA) purification system throughout the experiments. Fourier transform infrared-attenuated total reflectance spectroscopy (FTIR-ATR, Nicolet IS10, Thermo Fisher Scientific Inc., Waltham, MA, USA) analysis was performed to understand the functional groups of the adsorbent material and to monitor any changes in its structure that may occur after adsorption. In addition, scanning electron microscopy (SEM, Zeiss EVO LS10, Oberkochen, Germany) and X-ray diffraction (XRD. Malvern PANalytical X’Pert PRO, Malvern, UK) were used to analyze the adsorbent material exposed to different temperatures before and after adsorption to track any morphological changes that may have occurred in its structure. Samples were obtained from the solution medium at specific periods during the adsorption experiments, and these samples were analyzed in a UV–vis spectrophotometer (Shimadzu UV2600i, Osaka, Japan). The zeta potentials of SLM Stem-Natural and SLM Stem-800 °C adsorbents were measured at room temperature using a Brookhaven 90 Plus Zeta Sizer Analyzer. The samples were prepared in ultra-high distilled water with zero particle size at a concentration of 10 mg/mL.

### 3.2. Preparation of Adsorbent

**Figure 18:**
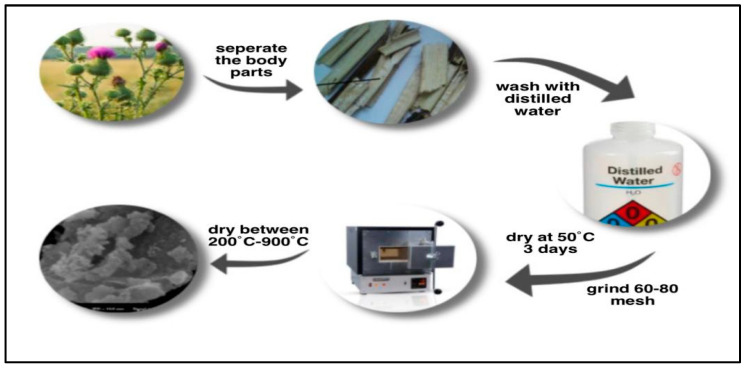
Figurative expression of the preparation procedure of the adsorbent from SLM Stem.

The collected SLM Stem parts were separated and washed several times with distilled water to remove any dust and water-soluble impurities. Then, they were dried in an oven at 50 °C for three days to eliminate any remaining moisture. The SLM Stem powder used as the adsorbent in this study was ground to a particle size of 60–80 mesh. Subsequently, adsorbents with different carbonization degrees were obtained by subjecting the SLM Stem powder to temperatures ranging from 200 to 900 °C using a muffle furnace. The procedure for preparing the adsorbent is shown in Figure 18.

### 3.3. Batch Adsorption Experiments

The studies on removing BB3 and BY28 hazardous dyestuffs with adsorbents derived from Silybum Marianum stem were explored with experiments using the batch method. During batch studies, 10 mL of dyestuff solution was taken into 50 mL beakers and the beaker was placed in a water bath with heating and shaking features. The dyestuff solution was prepared as a stock solution at a concentration of 1.0 g/L. Different concentrations of dyestuff solutions were diluted from this stock at appropriate ratios. Different concentrations of dyestuff solutions were obtained from this stock by dilution at proper proportions. The variables were: initial solution concentration 10–50 mg/L, adsorbent amount 0.01–0.20 g, initial pH value 2–12, agitation speed 100–200 rpm, contact time 5–50 min, and temperature 25–45 °C. Where necessary during the experiments, pH adjustments were made using 0.1 M HCl and 0.1 M NaOH solutions. Following the experimental protocols, the solution was centrifuged at 5000 rpm for 3 min to determine the concentration of dye solution in the supernatant. Meanwhile, UV-vis spectroscopy was performed at 654 nm for BB3 dye and 438 nm for BY28 dye. The results obtained were evaluated regarding the removal percentage (R%) and amount of adsorbed substance q_e_ (mg/g) at equilibrium, as in Equations (1) and (2) [69].

(1) $$Removal\;\left(\%\right)=\left[\frac{\left(C_{i}-C_{e}\right)}{C_{i}}\right]\times 100$$

(2) $$q_{e}=\frac{\left(C_{i}-C_{e}\right)\times V}{w}$$

In Equations (1) and (2), C_i_ and C_e_ represent the first and equilibrium concentrations (mg/L) of the used dyes, V represents the volume (L) of the dye solution, and w represents the mass (g) of the adsorbent.

### 3.4. Adsorption Isotherms

Some isotherms are employed throughout adsorption to investigate the interaction between the adsorbed substance and the adsorbent. These isotherms provide information about the type and capacity of adsorption. The most used isotherms are Langmuir and Freundlich. The equations for these curves are given as Equations (3) and (4), respectively.

(3) $$\frac{1}{q_{e}}=\frac{1}{K_{L}q_{max}}\times \frac{1}{C_{e}}+\frac{1}{q_{max}}$$

(4) $$Logq_{e}=LogK_{f}+\frac{1}{n}LogC_{e}$$

In Equation (3), q_max_ (mg/g) represents the maximum adsorption capacity and K_L_ (L/mg) indicates the Langmuir isotherm constant, which means the binding strength between the dye and the adsorbent material.

In Equation (4), K_f_ is the Freundlich constant used to measure the adsorption capacity. The value of 1/n in the same equation represents the adsorption intensity, where 0.1 ≤ 1/n ≤ 0.5 indicates a favorable adsorption process and 1/n ≥ 2 indicates an unfavorable adsorption process [72].

## 4. Conclusions

In this study, the stem part of the Silybum Marianum (SLM) plant, which is mainly known for the use of its seeds for therapeutic purposes in the literature, was used as an adsorbent. The adsorption performances of different carbonization products obtained by subjecting the SLM Stem plant powder to temperatures ranging from 200 to 900 °C were also examined by measuring their performances for removing the toxic dyes BB3 and BY28. As a result, it was observed that the removal percentage values of SLM Stem-Natural and SLM Stem-800 °C were higher than those of the adsorbents carbonized at other temperatures. The surface characteristics of the SLM Stem-Natural and SLM Stem-800 °C adsorbents were examined using IR, SEM, and XRD measurements. It was determined that the SLM Stem-Natural has a mainly non-porous material and SLM Stem-800 °C has a microporous structure. We looked at the optimum values of several parameters, including the initial dye solution concentration, contact time, temperature, pH, and agitation speed. For the BY28 dye, the following parameters were used: 0.05 g, 15 mg/L, 30 min, 40 °C, pH = 6, and 100 rpm for the SLM Stem-Natural adsorbent and 0.15 g, 30 mg/L, 30 min, 40 °C, pH = 10, and 150 rpm for the SLM Stem-800 °C adsorbent. For the BB3 dye, the SLM Stem-Natural adsorbent produced results of 0.20 g, 10 mg/L, 30 min, 25 °C, pH = 7, and 100 rpm and the SLM Stem-800 °C adsorbent produced results of 0.15 g, 15 mg/L, 40 min, 40 °C, pH = 10, and 100 rpm. A high removal potential for SLM Stem-800 °C, which successfully treated actual water by providing adsorption of around 99% in real-sample applications such as in tap water and Meriç (Evros) River water samples, was achieved. The reuse experiments conducted to shed light on the reuse of the adsorbent material showed that even after three cycles of regeneration, the removal percentage values of the adsorbent material could still reach around 90–99%. Different isotherms were subjected to adsorption data. Therefore, it was concluded that the Langmuir model was adequate for the adsorption process (r^2^ = 0.9987), and the best q_m_ value was 271.73 mg/g when the BY28 dye was used with the SLM Stem-800 °C adsorbent at 25 °C. As a result, it is understood that SLM Stem plant powders obtained from the Turkmenistan-Ashgabat region, which is an unexplored region of the world in this field, can be used for the removal of BB3 and BY28 toxic dyes from wastewater. Thus, it has been demonstrated that a natural substance, widely available in the area and often utilized as trash or animal feed, can be used to remove harmful BB3 and BY28 dyes. In this way, it is encouraging that it can be used to treat wastewater from the textile industry in a low-cost, efficient, and ecologically beneficial manner, especially in the concerned region and other similar places. It is also hoped that in the future, it will be a pioneer in exploring the adsorption capacities of different dyes, possibly even other harmful components, including heavy metals, in addition to the removal of the toxic BB3 and BY28 cationic dyes.

## Figures and Tables

**Table 1:** Chemical content of the SLM Stem-Natural.

Name of Analysis	Results	Applied Methods
Total protein	13.9 g/100 g ± 1.72	NMKL 6 [58]
Amount of polyunsaturated fatty acids	38.88% ± 0.08	Turkish food codex. [59]
Saturated fatty acids	25.87% ± 0.10	Turkish food codex. [59]
Monounsaturated fatty acids	16.49% ± 0.04	Turkish food codex. [59]
Trans fatty acids	18.76% ± 0.02	Turkish food codex. [59]
Carbohydrate content	68.93 g/100 g ± 0.13	FAO [60]
Total amount of fat	1.17% ± 0.08	AOCS (Am 5-04) [61]
Ash content	9.36% ± 0.03	ISO 5516 [62]
Humidity analysis	6.64% ± 0.02	ISO 1026 [63]

**Table 2:** Concentration of active groups on the surfaces of SLM Stem-Natural and SLM Stem-800 °C.

Name of Analysis	SLM Stem-Natural	SLM Stem-800 °C
Total basic sites (meq g^−1^)	0.61 ± 0.03	0.35 ± 0.01
Total acidic sites (meq g^−1^)	0.02 ± 0.01	0.001 ± 0.0002

**Table 3:** Langmuir and Freundlich isotherm parameters for the adsorption of BB3 and BY28 onto SLM Stem-Natural and SLM Stem-800 °C.

Langmuir	BB3 onto SLM Stem-800 °C	BB3 onto SLM Stem-Natural	BY28 onto SLM Stem-800 °C	BY28 onto SLM Stem-Natural
K_L_ (mg/L)	0.0811	0.0496	0.0129	0.0536
q_max_ (mg/g)	36.8053	13.9645	271.7391	36.5497
r^2^	0.9978	0.9720	0.9987	0.9897
Freundlich				
K_f_ (mg/L)	3.1314	0.7335	3.4516	2.2981
1/n	0.7289	0.7835	0.9743	0.7291
r^2^	0.9813	0.8593	0.9861	0.9863

**Table 4:** Comparison of the adsorption capacities of different adsorbents reported in the literature for the BB3 and BY28 dyes.

Adsorbent	Adsorption Capacity (mg/g)		Adsorption Model	References
Intercalated and tubular kaolinite	-	142.26	Freundlich	[12]
Kaolin	-	5.71	Langmuir	[15]
Macadamia seed husks	1.40	-	Langmuir-Freundlich	[21]
Powder activated charcoal (PAC)	151.30	-	Langmuir-Freundlich	[24]
Smectite rich natural clays (Ghassoul)	-	384.60	Langmuir	[25]
Chitosan-based adsorbent	166.50	-	Langmuir	[37]
Silybum marianum (SLM) Stem-Natural	13.96	36.54	Langmuir	[This study]
Silybum marianum (SLM) Stem-800 °C	36.80	271.73	Langmuir	[This study]

## Data Availability

Not applicable.
